# SpeSpeNet: an interactive and user-friendly tool to create and explore microbial correlation networks

**DOI:** 10.1093/ismeco/ycaf036

**Published:** 2025-02-24

**Authors:** Abraham L van Eijnatten, Luc van Zon, Eleni Manousou, Margarita Bikineeva, E R Jasper Wubs, Wim H van der Putten, Elly Morriën, Bas E Dutilh, L Basten Snoek

**Affiliations:** Theoretical Biology and Bioinformatics, Science4Life, Utrecht University (UU), Padualaan 8, 3584 CH Utrecht, The Netherlands; Theoretical Biology and Bioinformatics, Science4Life, Utrecht University (UU), Padualaan 8, 3584 CH Utrecht, The Netherlands; Theoretical Biology and Bioinformatics, Science4Life, Utrecht University (UU), Padualaan 8, 3584 CH Utrecht, The Netherlands; Theoretical Biology and Bioinformatics, Science4Life, Utrecht University (UU), Padualaan 8, 3584 CH Utrecht, The Netherlands; Department of Terrestrial Ecology, The Netherlands Institute of Ecology (NIOO), Droevendaalsesteeg 10, 6708 PB Wageningen, The Netherlands; Department of Terrestrial Ecology, The Netherlands Institute of Ecology (NIOO), Droevendaalsesteeg 10, 6708 PB Wageningen, The Netherlands; Department of Ecosystem and Landscape Dynamics (IBED-ELD), Institute for Biodiversity and Ecosystem Dynamics, University of Amsterdam, 1090 GE Amsterdam, The Netherlands; Theoretical Biology and Bioinformatics, Science4Life, Utrecht University (UU), Padualaan 8, 3584 CH Utrecht, The Netherlands; Faculty of Biological Sciences, Cluster of Excellence Balance of the Microverse, Institute of Biodiversity, Friedrich Schiller University, Jena 07745, Germany; Theoretical Biology and Bioinformatics, Science4Life, Utrecht University (UU), Padualaan 8, 3584 CH Utrecht, The Netherlands

**Keywords:** networks, microbiome, microbial ecology, interactive webtool, visualization

## Abstract

Correlation networks are commonly used to explore microbiome data. In these networks, nodes are microbial taxa and edges represent correlations between their abundances. As clusters of correlating taxa (co-abundance clusters) often indicate a shared response to environmental drivers, network visualization contributes to the system understanding. Currently, most tools for creating and visualizing co-abundance networks from microbiome data either require the researcher to have coding skills or are not user-friendly, with high time expenditure and limited customizability. Furthermore, existing tools lack a focus on the association between environmental drivers and the structure of the microbiome, even though many edges in correlation networks can be understood through a shared association of two taxa with the environment. For these reasons, we developed SpeSpeNet (Species-Species Network, https://tbb.bio.uu.nl/SpeSpeNet), a practical and user-friendly R-shiny tool to construct and visualize correlation networks from taxonomic abundance tables. The details of data preprocessing, network construction, and visualization are automated, require no programming ability for the web version, and are highly customizable, including associations with user-provided environmental data. Here, we present the details of SpeSpeNet and demonstrate its utility using three case studies.

## Introduction

The advent of high-throughput sequencing techniques and specifically amplicon-based sequencing has revolutionized microbial ecology [1, 2]. While research in this field used to be limited to culturable organisms, it is now possible to unravel the entire breadth of microbial diversity, the *microbiome*, of any environment. Moreover, recent advances also allow shotgun metagenome data to be used for accurate taxonomic profiling [3]. Microbiome sequencing techniques have become so accessible that data can now be generated at a faster pace than it can be analyzed, while bioinformatic analyses still often require substantial programming skills [4].

Amplicon sequencing is based on the targeted amplification of marker sequences directly from the environment [5, 6]. The observed sequences are grouped into either operational taxonomic units (OTUs) based on a percent similarity cutoff [7], or amplicon sequencing variants (ASVs) based on an error correction model that provides single nucleotide resolution [8]. The best-known marker sequence is the small subunit ribosomal RNA [5, 6, 9], which is suited for investigating bacterial and archaeal (16S) or eukaryotic (18S) communities. Another commonly used marker sequence is the internal transcribed spacer (ITS), suited for investigating the fungal community. These marker sequences are highly conserved and can therefore be used to assign taxonomy by mapping the sequence of each OTU/ASV to a reference database such as Silva [10], GTDB [11, 12], or other alternatives [13, 14].

The relationships between microbes are often studied and visualized using network analysis, especially correlation networks [15–20]. In such networks, nodes represent taxonomic lineages and edges represent correlations between them. When calculating correlations between taxa, two important characteristics of amplicon sequencing data need to be taken into account: “Compositionality” and “sparsity” [21–25]*.* Amplicon data are compositional because the total read-count per sample is dependent on sequencing read depth and stochastic polymerase chain reaction (PCR) amplification. As a result, the data only show the relative abundance of taxa, and not their absolute abundance [21, 22]. Correlations calculated between relative abundances can be artefactual. For example, an increase in the absolute abundance of one taxon will cause a decrease in the relative abundance of all other taxa, resulting in spurious correlations. The possible bias caused by compositionality can be mitigated using specialized normalization methods such as the centered log-ratio (CLR) transform or network inference methods such as SparCC [18, 26, 27]. A second important characteristic of amplicon sequencing data is sparsity [23–25]. Many microbes are exceedingly rare, such that they exist around or below the detection limit of the sequencing experiment [28–30]. Hence, zero counts can mean either true absence of taxa from the sample or presence below the detection limit. False zero counts can also cause spurious associations between rare taxa, because variation in abundance patterns below the detection limit is missed. This bias can be mitigated by zero imputation and taxa filtering.

Correlations between the relative abundance of microbial taxa usually indicate similar responses to changes in the biotic or abiotic environment rather than direct interactions between taxa (such as cross-feeding, predation, competition, etc.). Hence, clusters of taxa in microbial correlation networks (co-abundance clusters) often result from a shared response to one or more environmental drivers [23]. Incorporating environmental factors and identifying and interpreting co-abundance clusters are important open challenges in microbiome network science (challenges 4 and 8 in [24]). Despite the generally high impact of environmental drivers on the structure of the microbiome, most network-visualization tools do not place adequate emphasis on the association between co-abundance clusters and environmental drivers. Some existing approaches, such as Flashweave and CoNet, incorporate environmental factors as nodes in the network [31, 32]. However, the association between taxa, co-abundance clusters, and environmental factors can be investigated and visualized more precisely by overlaying each node in the network with its association with environmental factors instead. This also allows for using different quantitative metrics for the correlation network and the associations with environmental factors.

Current microbiome network tools are often inaccessible or impractical for many researchers. Microbiome networks may be constructed using general-purpose tools and R packages such as network [33], igraph [34], Cytoscape [35], or Gephi [36]. R-based packages for making networks specifically from microbiome data include NetCoMi [37], microeco [38], and ggClusterNet [39]. However, these still require programming skills, and it is often difficult to adjust the details and focus of the visualizations. The galaxy-based iNAP [40] does not require any programming skills but is inconvenient to work with due to the limitations of the galaxy platform and the lack of customization of the networks. The CoNet app [32] lacks the capacity for quick exploration, interactivity, and making co-abundance clusters. Taken together, there is currently no user-friendly, interactive software to make correlation networks and clusters from OTU/ASV tables and visualize environmental drivers of the microbiome.

To enable more researchers to make, customize, and use networks in their investigations, we introduce SpeSpeNet (Species-Species Network, https://tbb.bio.uu.nl/SpeSpeNet), an R-shiny-based web-application that makes correlation networks and visualizes them directly from taxonomic abundance tables. SpeSpeNet accelerates discovery and facilitates researchers by automating and customizing the data preprocessing, construction of the network, and network visualization. The taxa in the network can be colored by taxonomy, by association with user-defined environmental parameters, or by co-abundance clusters. SpeSpeNet provides options to take compositionality and sparsity into account (see the Discussion for an in-depth reflection on these topics). Apart from correlation networks, SpeSpeNet also uses other visualization techniques such as traditional stacked taxonomy barplots, and boxplots showing the association between co-abundance clusters and environmental parameters. Here, we present the details of the SpeSpeNet tool and demonstrate its utility in microbial ecology with three case studies.

## Materials and methods

### Dependencies

SpeSpeNet is an R-shiny tool, developed with the shiny package [41]. The layout of the app is generated using the “cyborg” option from the shinythemes package [42]. Network visualizations are made with the igraph package [34], using the ggraph package [43] for programmatic convenience. The SparCC algorithm [44] is run using the SpiecEasi [45] implementation. For other plots, SpeSpeNet uses the ggplot2 package [46], supplemented by the ggdark package [47]. SpeSpeNet uses the MGnifyR package [48] to download public data from the MGnify database into SpeSpeNet. The network and other plots are made interactive with the ggiraph package [49] and the plotly package [50], respectively. The code makes use of the tidyverse [51] and tidygraph [52] packages for convenience.

### Workflow

**Figure 1:**
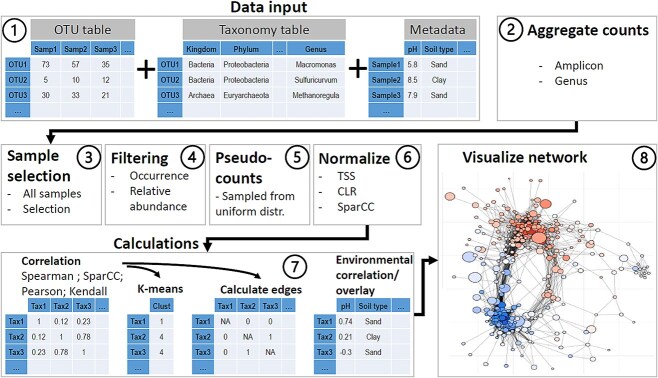
Consecutive steps of the SpeSpeNet workflow. (1) The user uploads the count table, taxonomy table, and environmental metadata table. (2) The user can choose to aggregate the counts at the genus rank. (3) The user can choose to use all samples or a selection. (4) Next, taxa are filtered on zero counts and relative abundance with user-specified cutoffs. (5) By default, zero counts are substituted with random pseudo-counts below the detection limit to prevent spurious correlations. (6) The data are normalized using TSS or CLR transformation. (7) Calculation of correlation matrix (Spearman, SparCC, Pearson, or Kendall), clusters, and environmental overlay. (8) Network is visualized.

SpeSpeNet uses an automated workflow to generate correlation networks from count tables using user-specified parameters (Fig. 1).

### Data format, aggregation, and selection (Step 1:3)

Data can be input as a phyloseq object (.rds file) or as three separate tab delimited .txt files that contain the OTU/ASV table, the taxonomy table, and the environmental table. For the details on the data input format, see the SpeSpeNet manual (Supplementary text S1). SpeSpeNet can also download studies from the MGnify database if provided with an MGnify study accession number.

The user can choose to aggregate the OTUs/ASVs to genus rank. In this case, the read counts of all taxa belonging to the same genus are summed before normalization and the nodes in the network will be genera instead of OTUs/ASVs. Taxa that cannot be determined at the genus rank are aggregated to the deepest known taxonomic rank. As an example, all taxa that can only be determined to the order *Burkholderiales* will be a single node in the network, possibly containing multiple genera and families. By default, SpeSpeNet will make a network using all samples provided in the data uploaded. The user can also choose to make a subnetwork using categorical variables in the metadata. After the user selects a categorical variable, SpeSpeNet lists all the categories in this variable that correspond to at least eight samples. After the user selects a category, SpeSpeNet will recalculate the network generation process from Step 4 using only samples from the selected category.

### Data processing (Step 4:6)

Next, the taxa are filtered using two user-specified thresholds: (i) The minimum number of samples taxa need to occur in (have nonzero read counts) and (ii) the minimum % of total reads taxa need to have in at least one of the samples. Only OTUs/ASVs/genera that satisfy both thresholds are included in the network. After filtering taxa, the remaining zero values are substituted with random pseudo-counts from a uniform distribution between 0.1 and 1 by default. Alternatively, the user can use other zero replacement methods outside of SpeSpeNet and upload the imputed count table. In this case, a constant pseudo-count of 1 is added if CLR-normalization is selected or no pseudo-counts if total sum scaling (TSS) normalization is selected. Next, the read counts are normalized using TSS or CLR-transformation. If SparCC is selected the normalization is done by the algorithm.

### Network construction and visualization (step 7:8)

The filtered matrix with normalized abundances is used to calculate the correlation matrix with dimensions taxa-by-taxa. The default correlation method is Spearman, but SpeSpeNet also allows the user to choose Pearson or Kendall correlations or to infer the correlations using the SparCC algorithm.

SpeSpeNet then constructs a graph from the correlation matrix using the function graph.adjacency() from the igraph package. All edges between taxa with a correlation lower than the specified cutoff are deleted using the function delete.edges(). By default, SpeSpeNet uses only positive correlations to construct edges. We chose this approach because co-occurring microbes will have positive correlations with taxa in the same cluster but negative correlations with taxa in different clusters. Hence, including negative correlations in the network can obscure environment-driven clustering present in the network. SpeSpeNet does have the option to include negative correlations as edges, but we only recommend this for datasets with a homogeneous environment between samples such as human microbiomes. If this option is chosen, negative correlations are shown as orange and positive correlations are shown as blue. After constructing the edges, the network is plotted using the ggraph() function from the ggraph package. The size of the nodes in the network is proportional to the square root of the mean relative abundance over all samples of the corresponding taxa. The user can choose whether to plot isolated taxa (nodes without edges). However, these taxa are still used in the *k*-means clustering. The user can also set the curvature, width, and transparency of the edges in the network.

The network can be colored on (i) *k*-means cluster assignment, (ii) association with environmental variable, and (iii) taxonomy of the nodes. In Case (i), the *k*-means algorithm is run on the taxa-by-taxa correlation matrix with a user-specified *k* (number of clusters). The assigned clusters are then used to color the network. In Case (ii), the environmental variable can either be numeric or be categorical. For a numeric environmental variable, the Pearson correlation between the normalized abundance and the environmental variable is calculated for each taxon and used to color the network. NA values are discarded when calculating the Pearson correlations. For a categorical environmental variable, SpeSpeNet calculates the category in which each node has the highest mean normalized abundance. This is then used to color the network. Finally, in Case (iii) a user-specified taxonomic rank is used to color the nodes of the network. The 15 clades with the highest relative abundance are shown, and all other taxa are labeled as “Other” to constrain the size of the legend and the number of colors in the network.

### Summary tab

The summary tab shows two plots. The first is a barplot of the taxonomic composition at a chosen taxonomic rank. If the network is colored on a categorical environmental variable, the barplot will show the taxonomic composition per category. If the network is colored on *k*-means cluster, the barplot shows the taxonomic composition of each cluster separately. In this case, the user can choose whether they want to show the mean relative abundance as a percentage of the cluster or as a percentage of all reads. Finally, if the network is colored by taxonomy or a continuous environmental variable in the network tab, the barplot shows the mean relative abundance per clade over all samples.

The second plot in the summary tab is a boxplot of the Pearson correlations between the normalized abundances of the taxa in the network with a continuous environmental variable of choice. If SparCC was used to construct the network, CLR-transformed abundances are used for the environmental correlations. When the network is colored on *k*-means, the boxplot is faceted on cluster.

### Downloads and customization

The SpeSpeNet network images can be downloaded as .png files by clicking on the blue arrow in the top-right corner of the network visualization. To facilitate customization of the network visualizations in R, users can download the network as a tidygraph object in the Download tab. Using the script Plot_from_tidygraph_object.R (available on github), users can make their own personalized network visualizations from the downloaded tidygraph objects. The node metadata, edges, and correlation matrix can also be downloaded separately from SpeSpeNet as .txt files.

## Results

### SpeSpeNet correlation networks

**Figure 2:**
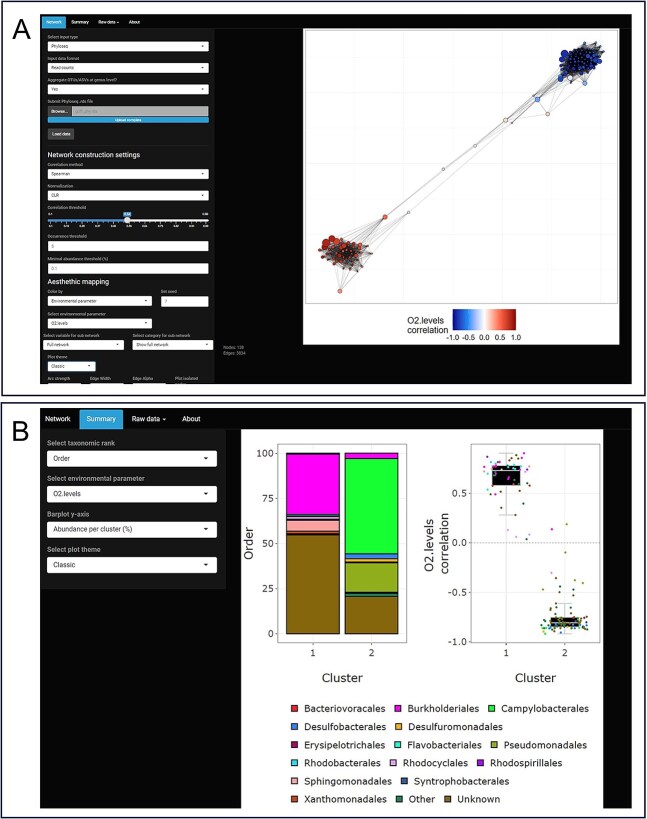
Screenshots of the network and summary tabs of SpeSpeNet. Same data as in case study 1 [53]. (A) The network tab shows a microbiome network where every taxon is colored on its correlation with O_2_ levels. (B) The summary tab shows a barplot of the taxonomic composition at order rank and boxplots with the correlation with O_2_ for two clusters of taxa.

SpeSpeNet (Species-Species Network) is a fast, user-friendly, and comprehensive tool for visualizing and analyzing microbiome data using correlation networks. SpeSpeNet has two tabs, each visualizing distinct aspects of the dataset: the network tab (Fig. 2A) and the summary tab (Fig. 2B).

**Figure 3:**
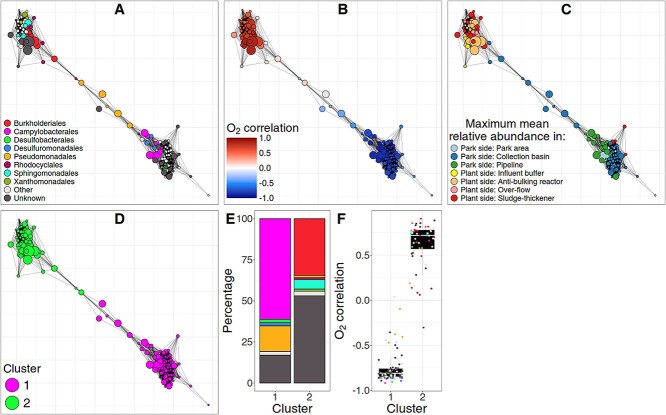
SpeSpeNet co-abundance network of the microbiome in a poly-contaminated city park and the corresponding water treatment plant [53]. Nodes are genera and edges are SparCC correlations > 0.63. (A) Network colored by taxonomic annotation at the order rank. (B) Network colored by Pearson correlation with O_2_ levels. (C) Network colored by the sampling site in the park/remediation pipeline in which genera were most abundant. (D) Network colored by *k*-means cluster assignment with *k* = 2. (E) Barplot of taxonomic composition at order rank for the co-abundance clusters from (D) (colors as in (A)). (F) Distribution of correlation values with O_2_ for the genera in the clusters from (D) (colors as in (A)).

In the network tab, SpeSpeNet displays a correlation network where nodes are taxa and edges are correlations with a user-specified method and cutoff. We used a study on the microbial community in a bioremediation pipeline [53] (discussed in more detail in the next section) to showcase the networks made by SpeSpeNet. To explore the association between taxonomy and environmental drivers in the network, SpeSpeNet can color the nodes using four types of variables: (i) the taxonomy of the nodes at a chosen rank (e.g. order) (Fig. 3A); (ii) for a continuous environmental variable (e.g. O_2_), its correlation with the nodes (Fig. 3B); (iii) for a categorical environmental variable (e.g. location in the pipeline), the categories where nodes have the highest relative abundance (Fig. 3C); and (iv) the nodes’ co-abundance clusters, detected based on customizable settings (Fig. 3D). The network is interactive: it shows the full taxonomy when the mouse is hovered over a node. Furthermore, SpeSpeNet can use categorical variables in the metadata to make a subnetwork based on a subset of the samples. This subnetwork feature allows the researcher to compare community structure and environmental drivers in contrasting environments.

In the summary tab, SpeSpeNet offers an extended view of the clusters/categories in the network in terms of the taxonomic composition and the association with environmental drivers. This is achieved using two figures: (i) a barplot of the taxonomic composition of clusters or categories in the network (Fig. 3E) and (ii) a boxplot of the correlations of the taxa abundance in each cluster or category with a selected environmental variable (Fig. 3F).

SpeSpeNet provides many options to customize network generation in terms of the input format, network parameters, and aesthetic layout. Data can be input as phyloseq objects [54] or .txt files (see Supplementary text S1 for details on the required data format) or can be downloaded from the MGnify database [55] into memory by SpeSpeNet. SpeSpeNet provides options to filter the taxa based on presence/absence and relative abundance thresholds. Additionally, various network options can be specified: (i) The normalization method (TSS or CLR); (ii) The sparsity method (imputing random pseudo-counts below the detection limit [23, 56] or user performing zero imputation before upload); (iii) the type of correlation (Spearman, SparCC, Pearson, or Kendall) used to calculate the correlations between taxa; (iv) the correlation cutoff that defines the edges in the network; (v) whether to use only positive or positive and negative correlations to define edges; and (vi) the network clustering algorithm and parameters. Furthermore, SpeSpeNet lets the user specify the aesthetics of the network (Supplementary Fig. S1): width, transparency and arc of edges, the color of the background, and whether to plot isolated nodes (nodes without edges). A new network is automatically generated when any of these options are changed.

To demonstrate the utility of SpeSpeNet we present three case studies. First, we investigate a dataset about the microbial community in a hydrocarbon-contaminated park and the associated treatment plant [53]. Second, we explore a dataset of marine microbiome samples from the north-western Mediterranean (Blanes Bay, Spain) to investigate phytoplankton blooms [57]. Finally, we used SpeSpeNet to analyze a dataset about the effect of different organic amendments on the soil microbiome and microbial functional gene abundance [58].

### Case study 1: microbial community in a poly-contaminated park and the associated treatment plant

We investigated the microbial community in a hydrocarbon-contaminated park and the associated treatment plant [53]. The samples came from seven different stages of the bioremediation process. These samples could be subdivided into anaerobic soil samples, representing the “park-side” of the system (Stages 1–3) and samples from the aerated water treatment “plant side” (Stages 4–7). The network made by SpeSpeNet clearly recapitulated two types of microbiomes as separate co-abundance clusters: one dominated by taxa of the order *Burkholderiales*, the other dominated by taxa of the order *Campylobacterales* (Fig. 3A).

We used SpeSpeNet to investigate potential environmental drivers of the two co-abundance clusters visible in the network. Coloring the taxa based on their Pearson correlation with oxygen (O_2_) showed that the two clusters in the network are correlated with O_2_ levels (Fig. 3B). This result aligned with the conclusions by Hauptfeld *et al*. [53]*:* in the water treatment plant, the added O_2_ facilitated the change from an anaerobic to an aerobic microbial community. The top-left cluster consisted exclusively of microbes that correlated positively with O_2_ levels (aerobic community), whereas the bottom-right cluster consisted of microbes that correlated negatively with O_2_ levels (anaerobic community). SpeSpeNet further showed that the taxa in the anaerobic community were most abundant in the park-side stages, whereas the taxa in the aerobic community were most abundant in the treatment plant-side stages (Fig. 3C).

The two different communities were distinguished using *k*-means clustering (Fig. 3D), after which the taxonomic composition of the two communities could be visualized (Fig. 3E). The barplots showed that more than half of the park-side community consisted of taxa belonging to the orders *Campylobacterales* and *Pseudomonadales*, while most of the plant-side community consisted of taxa belonging to the orders *Burkholderiales* and *Sphingomonadales*. Finally, SpeSpeNet visualized the distribution of Pearson correlation with O_2_ levels in each community in a boxplot (Fig. 3F), confirming their inverse correlation with O_2_ levels.

### Case study 2: phytoplankton-blooms in the north-western Mediterranean

As a second case study, we used a dataset of marine microbiome samples from the north-western Mediterranean (Blanes Bay, Spain) [57]. In this study, the surface water was sampled monthly for 10 consecutive years and the microbial community composition was determined using the 16S and 18S marker genes. The 16S and 18S tables were merged after rarefaction to create a single abundance table, needed to calculate correlations between all taxa. Several environmental factors were also measured, including temperature and total chlorophyll a. Chlorophyll a is considered a proxy for the phytoplankton biomass [59] and it peaks during phytoplankton blooms [60]. We studied the taxa involved in phytoplankton blooms in Blanes Bay by correlating taxa relative abundance with chlorophyll a.

**Figure 4:**
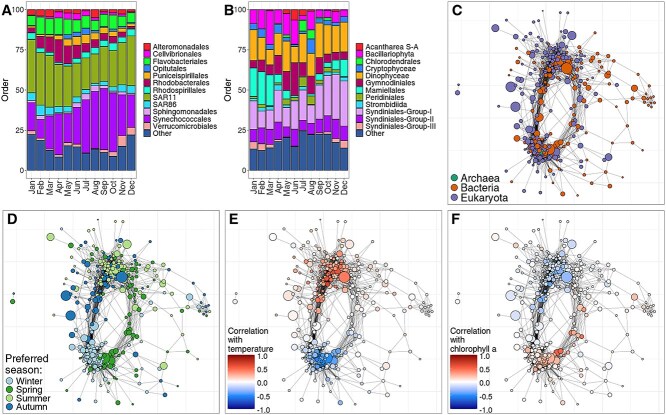
Inter-kingdom associations in a 10-year longitudinal study in the north-western Mediterranean [57]. The nodes are genera and edges represent Spearman correlations > 0.37 between TSS normalized abundances. (A) Taxonomic composition of the prokaryotic community per month at order rank. (B) Taxonomic composition of the eukaryotic community per month at order rank. (C) Co-abundance network colored by the kingdom of each genus. (D) Network colored by the season during which taxa have the highest mean relative abundance. (E) Network colored by the correlation between each genus and surface water temperature. (F) Network colored by the correlation between each genus and chlorophyll a level.

First, we sanity checked the data using SpeSpeNet by making barplots of the prokaryotic and eukaryotic microbiome in each month and found previously identified seasonal patterns from the area, such as the increase in *Synechococcales* between July and October [61] (Fig. 4A) and the strong seasonality of the *Mamiellales* [62] (Fig. 4B). Next, we used SpeSpeNet to make an inter-kingdom correlation network using the merged 16S and 18S abundance tables (Fig. 4C). Because the samples varied in time but not in space, the correlations showed a cyclical structure that corresponded to the changing of environmental conditions such as temperature and nutrient levels, during the passing of the seasons (Fig. 4D and E and Supplementary Fig. S2).

Next, we colored the network by the correlation with chlorophyll a (Fig. 4F). Mousing over nodes with high correlations with chlorophyll a showed that some belonged to the families *Roseobacteraceae* [63] and *Flavobacteriaceae.* These two families have been identified as the two main bacterial groups associated with phytoplankton blooms [64]. To quantify the associations between taxa relative abundance and chlorophyll a levels, we used the spring and summer samples to calculate Pearson correlations. Out of the six prokaryotic genera with the highest correlations with chlorophyll a, five belonged to *Roseobacteraceae* (*Amilybacter*, *Loktanella*, and *Planktomarina*) and *Flavobacteriaceae* (*Aurantivirga* and *Winogradskyella*).

In the case of *Roseobacteraceae*, the association with phytoplankton blooms was previously linked to cross-feeding interactions with micro-algae such as diatoms involved in phytoplankton blooms [65, 66]. The top three eukaryotic genera with the highest correlations with chlorophyll a were diatoms (*Pseudo-nitzschia*, *Thalassiosira*, and unknown *Coscinodiscophyceae*). Members of *Roseobacteraceae* have been found in the microbiome of various diatoms including *Pseudo-nitzschia* [67] and *Thalassiosira* [68]. Therefore, we used SpeSpeNet to identify *Roseobacteraceae* genera that strongly correlated with the relative abundance of these two diatom genera (Spearman correlation > 0.6). We found that *Planktomarina* and *Amylibacter* strongly correlated with *Pseudo-nitzschia* (*ρ* = 0.68 and *ρ* = 0.62, respectively). Potentially, bacteria of *Planktomarina* and/or *Amylibacter* could be involved in metabolic interactions with *Pseudo-nitzschia* diatoms. This case study about phytoplankton blooms shows that SpeSpeNet captures cyclic seasonal dynamics and can be combined with a priori knowledge to form hypotheses about microbial interactions.

### Case study 3: influence of organic amendments on the soil microbiome and greenhouse gas emissions

**Figure 5:**
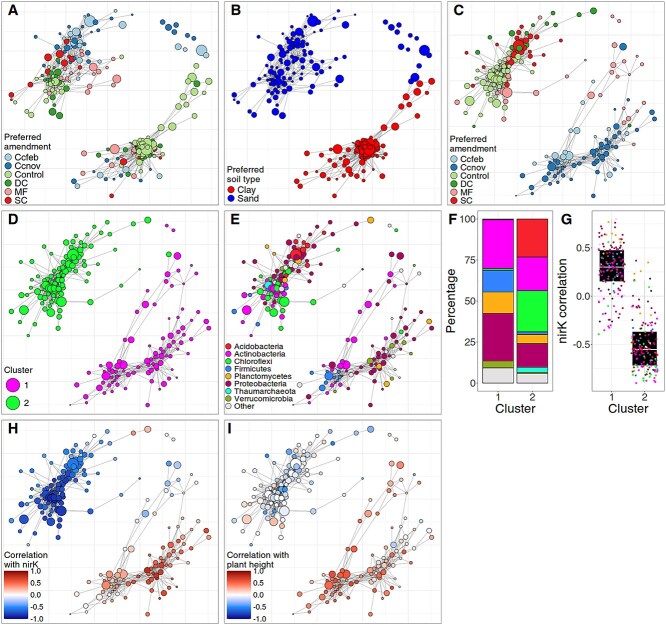
Effect of amendments on the microbiome in a mesocosm experiment [58]. In all panels, nodes are genera and edges are Spearman correlations > 0.72 between CLR-transformed abundances. (A) Network using all samples, colored on the amendment condition in which taxa have the highest CLR-transformed abundance. (B) Same network as in (A) colored on the soil type in which genera have the highest mean CLR-transformed abundance. (C) Subnetwork using only clay samples, colored by the amendment condition in which taxa have the highest mean CLR-transformed abundance. (D) Subnetwork colored by *k*-means clustering with *k* = 2. (E) Subnetwork colored by taxonomy at phylum rank. (F) Barplot of the taxonomic composition at phylum rank of the two co-abundance clusters shown in (D). (G) Distribution of the correlation with the qPCR abundance of the *nirK* genes for the genera in the co-abundance clusters shown in (D). (H) Subnetwork colored by correlation of CLR-transformed abundance of genera with the qPCR abundance of the *nirK* gene (involved in NO_2_ reduction). (I) Subnetwork colored by correlation of CLR-transformed abundance of genera with the plant height after the experiment.

For the third case study, we use a dataset about the effect of different organic amendments on the soil microbiome and microbial functional gene abundance [58]. Six different amendments were applied to sand and clay soils: Residue derived from cover crops harvested in February (Ccfeb) or November (Ccnov), a mixture of digestate and compost (DC), a mixture of sewage sludge and compost (SC), mineral fertilizer (MF), and a control (Control). Half of the plots were planted with *Triticum aestivum* (common wheat). We used SpeSpeNet to make a network of the soil microbiome and colored by the treatment in which taxa have the highest mean normalized abundance. We noticed two main clusters in the network, both with a mixture of microbes favoring different amendments (Fig. 5A). Using the soil type to color the network showed that the two clusters corresponded to microbes favoring sand or clay soils (Fig. 5B). To reveal the effect of the amendments on the microbiome through the confounding effect of soil type, we used SpeSpeNet to generate a subnetwork using only the samples annotated as clay.

We created a subnetwork to investigate the effect of amendments on the microbiome in clay soils (Fig. 5C). SpeSpeNet shows two co-abundance clusters: One cluster contains all taxa that favor the Control, DC, and SC amendments (control cluster) and the other cluster contains almost all taxa that favor the Ccfeb and Ccnov amendments (cover crop cluster). Taxa that favor the MF amendments are more spread between the co-abundance clusters. This shows that especially the cover crop-based amendments favor distinct taxa compared to unamended soils. To further investigate the differences in microbial community between the co-abundance clusters, we detected the co-abundance groups using the *k*-means algorithm (Fig. 5D) and plotted the respective taxonomy (Fig. 5E and F). The taxonomy differs strikingly between the co-abundance clusters, even at phylum rank, with the cover crop cluster being enriched for *Proteobacteria* and *Firmicutes* but depleted for *Acidobacteria* and *Chloroflexi*.

The dataset also contained quantitative PCR (qPCR) abundances of various functional marker genes. We wondered whether the different taxonomic compositions of the two co-abundance clusters translated to different community functions. Therefore, we investigated whether the taxa in the two co-abundance clusters had different correlations with the qPCR abundances of functional genes related to nitrogen cycling. Various nitrogen cycling genes such as *NirK* (NO_2_ reduction) (Fig. 5G and H), *NifH* (nitrogen fixation) (Supplementary Fig. S3A), and *NOSZII* (N_2_O reduction) (Supplementary Fig. S3B) are strongly associated with the two clusters in the network. This could indicate that the co-abundance clusters in the network are largely driven by the impact of amendments on nitrogen availability in the soil. Interestingly, a subset of the microbes in the cover crop cluster, which positively correlates with increased nitrogen cycling genes, has strong positive correlations with plant height (Fig. 5I). Mousing over the network in SpeSpeNet shows that these include known plant beneficial nitrogen-fixing genera such as *Lysobacter* (Supplementary Fig. S4A) and *Achromobacter* (Supplementary Fig. S4B). In this case study, we show how SpeSpeNet can be applied to rich datasets with different data types to quickly form hypotheses about the interplay between the environment, microbiome, microbial function, and other biota.

## Discussion

Here, we present SpeSpeNet, an interactive network visualization tool that facilitates visualization and investigation of co-abundance networks by streamlining the network construction process from taxonomic abundance tables. With three different use cases, we showed how these networks allow discovery of meaningful microbiome-environment associations. The networks are highly customizable in terms of the network parameters and aesthetics. SpeSpeNet provides options to correct for bias introduced by sparsity and compositionality. Furthermore, network construction only takes a few seconds, even for large datasets. All of this can be done at the click of a button and requires no programming skill on the part of the user.

Currently, several alternative methods to SpeSpeNet also construct networks from microbiome data. For example, the R-package NetCoMi [37] handles data filtering, normalization, and network construction. We used case study 1 to compare NetCoMi with SpeSpeNet. While SpeSpeNet and NetCoMi were able to accurately cluster the microbiome (Fig. 3A and Supplementary Fig. S5A), NetCoMi lacked options to overlay the network with taxonomy or environmental associations. Taxonomic (Supplementary Fig. S5B) or environmental (Supplementary Fig. S5C) overlays for NetCoMi networks can be scripted by the user but there are no integrated, interactive options for achieving this. Therefore, creating informative overlays in NetCoMi requires the researcher to have extensive programming ability. Furthermore, the network visualizations are not of the same aesthetic quality as those generated by SpeSpeNet (Supplementary Fig. S5). Other R-based approaches such as microeco [38] and ggClusterNet [39] have similar limitations. As far as we know, the only other approaches that require no programming from researchers are the galaxy-based iNAP [40] and the Cytoscape-based CoNet [32]. SpeSpeNet improves on these methods because of its interactivity, quick explorative capabilities, improved integration of environmental factors, and aesthetic visualizations, making SpeSpeNet a valuable addition to the current microbiome network analysis approaches.

SpeSpeNet offers two ways to normalize the input data for use with standard Pearson, Spearman, or Kendall correlations. The first option is TSS, where the counts are transformed into relative abundances by normalizing by the total read depth of the sample. This normalization is commonly used, but TSS normalization can induce spurious associations between taxa, because relative abundances cannot vary independently from each other. In practice, the bias introduced by compositionality is minor for datasets with a large number of taxa, as found in, e.g. soil or marine microbiomes. In these cases, TSS normalization will not introduce many spurious edges into the network [44]. The second normalization method offered by SpeSpeNet is the CLR transform. The CLR transform is part of the family of log-ratio transformations, which also includes the additive-log ratio transformation and the isometric-log ratio transformation. Such transformations remove the sum constraint from compositional data, mapping it to Euclidean space [26, 27]. Out of these transformations, the CLR transform has the clearest interpretation. Moreover, a recent preprint showed that the CLR transform effectively mitigates the compositionality of the data if the number of taxa is larger than 100 [69], which is almost always the case for microbiome data. For these reasons, SpeSpeNet offers CLR normalization to account for the bias introduced by compositionality. We recommend users choose CLR normalization over TSS normalization for datasets with lower diversity.

SpeSpeNet also offers the SparCC algorithm for inferring correlations [44]. SparCC infers more accurate correlations than standard correlation methods on TSS or CLR normalized data [44], but comes at the expense of higher runtime. SparCC approximates linear Pearson correlations between the log-transformed taxa. This approximation is based on two assumptions: (i) there is a sufficient number of taxa and (ii) most taxa are not strongly correlated with each other. Whether standard correlation or SparCC is most appropriate thus depends on the dataset. For example, for soil microbiome datasets standard correlations will be most appropriate. Such datasets have very high diversity, and therefore the bias introduced by compositionality is very minor [18, 44]. At the same time, assumption (ii) of the SparCC algorithm is likely to be violated in soil environments, because large environmental gradients in, e.g. pH or moisture can cause many true correlations between taxa. For less diverse environments such as the human gut, the bias introduced by compositionality is larger due to lower diversity. Furthermore, assumption (ii) from the SparCC algorithm is likely to hold in the human gut, because the environment is much more constant between samples/individuals, and may not drive as many correlations between taxa. Therefore, the use of SparCC for correlation inference is strongly recommended for low diversity, homogeneous environments like the human gut [18, 23].

Apart from SparCC, many additional network inference methods have been developed [31, 70–73]. Some network inference methods, including SpiecEasi and Flashweave [18, 31], attempt to infer direct interactions between taxa or taxa and the environment by leveraging the concept of conditional independence [26]. We did not implement such network inference methods in SpeSpeNet because they are not well-validated or computationally feasible (for an interactive tool) for diverse microbiomes such as found in soil or marine environments, and because different methods give different results [18] despite strong claims about the interpretation of edges. Instead, we implemented SparCC, a method that infers compositionally aware correlations, along with traditional correlation metrics including Spearman, Pearson, and Kendall. Such correlations indicate co-occurrence, but cannot be used as evidence of ecological interactions. Focusing on co-occurrence rather than direct interactions prevents over-interpretation of edges and allows SpeSpeNet to put emphasis on the association between the general correlation structure and the environment.

Sparsity due to presence of taxa below the detection limit can lead to spurious correlations between taxa. In the case of rank-based correlations such as Spearman (generally preferred because rank-based correlations do not assume linear relationships between taxa), many zero values cause rank-ties, inducing spurious correlations. How to deal with this sparsity has not yet been adequately addressed for microbiome data [24, 25]. One approach is to impute estimated counts for zero values based on the nonzero counts. Various general purpose zero-replacement methods have been developed for compositional data [74–76], including specifically for microbiome data [77, 78]. However, existing methods are either unstable for highly sparse data [79] or make assumptions about taxon distributions that may not be congruent with real world datasets [80]. In the absence of a consensus, more conservative approaches such as taxon filtering or adding random pseudo-counts are more appropriate [23, 56]. SpeSpeNet offers options to filter sparse taxa based on zero counts or low abundance. Note that taxa filtering can cause its own bias and must be done with care, because taxa that prefer under-represented habitats are more likely to be filtered out [24]. By default, SpeSpeNet substitutes the remaining zero values with random pseudo-counts below the detection limit. As an alternative to random pseudo-counts, the user has the option to use other zero replacement methods outside of SpeSpeNet and upload the imputed count table. Next to the filtering options and pseudo-counts, CLR normalization can alleviate some of the sparsity bias for moderate levels of sparsity [69]. Finally, SpeSpeNet has the option to aggregate taxa at the genus rank, resulting in lower sparsity compared to amplicon level counts at the expense of taxonomic resolution.

SpeSpeNet visualizes associations between the microbiome and the environment by correlating taxa with environmental variables. Moreover, SpeSpeNet shows the distribution of these correlations for the identified co-abundance clusters, pinpointing potential drivers of the microbiome. We stress that such patterns, while they can be insightful, always need to be interpreted with caution. After all, environmental variables are often highly (anti-)correlated with each other, such that even if only one variable is driving the microbiome, many different variables will show a similar, visually convincing pattern (Supplementary Fig. S6). Furthermore, correlations cannot inform on the direction of the effect and if the microbiome is driving the environment, or the environment is driving the microbiome cannot be inferred from correlations. Thus, SpeSpeNet should be used to explore and generate hypotheses rather than conclusions. These hypotheses can be further investigated using appropriate techniques and statistics and inform or inspire targeted follow-up experiments.

Edges in the networks generated by SpeSpeNet should also be interpreted with caution, for two major reasons. Firstly, the correlation-cutoff defining an edge is to some extent arbitrary. Two taxa may have correlations that are barely higher than the cutoff, whereas two other taxa may have correlations that are just lower. The network will show an edge between the first two taxa but not the second two, despite the correlations being almost equal (although in either case they will likely end up in the same co-abundance cluster through correlations with other taxa). In other words, the discrete nature of the network does not reflect the continuous nature of the correlation matrix. Secondly, researchers should exercise caution when ascribing an ecological interpretation to edges in correlation networks. Edges indicate, per definition, that taxa correlate in their abundance pattern. This rarely implies a direct interaction in the ecological sense, such as predation or cross-feeding [81]. Instead, taxa often simply respond in a similar way to external drivers, causing their relative abundances to change in similar directions, e.g. in response to a fluctuating environment. Alternatively, edges could result from a shared response to changes in community composition and its functional potential. Either scenario could cause a correlation between the relative abundance of two taxa without requiring a direct ecological interaction between them. Such patterns are interesting as well, because how microbes respond similarly to environmental changes is highly relevant for topics such as the effect of temperature, floods or drought on the soil microbiome, or the impact of a diet/drug on the gut-microbiome.

SpeSpeNet uses *k*-means on the correlation matrix to identify communities. This comes with several caveats because the *k*-means algorithm is not explicitly designed for community detection. First, the *k*-means algorithm assigns all taxa to one of the clusters, regardless of whether a taxon has a clear co-abundance pattern with any of the other taxa. Secondly, *k*-means assumes spherical clusters and cannot accurately detect communities with a more complex configuration in correlation-space. Care must also be taken when assigning a biological interpretation to the *k*-means clusters. For example, a user might interpret a *k*-means cluster as a group of microbes being driven by similar environmental drivers based on the boxplot in the summary tab. However, the *k*-means clusters can include outlier taxa that correlate differently with environmental data than others (as in, e.g. Fig. 5G cluster 1). If these outlier taxa happen to have a very high relative abundance, they will have a strong impact on the taxonomic composition of the *k*-means clusters (as shown in the barplots in the summary tab). In such cases, the user must take care not to interpret this change in taxonomic composition as being caused by or responsible for the environmental pattern. We re-emphasize that SpeSpeNet is a tool for ecological data visualization and hypothesis generation.

In the future, SpeSpeNet could be extended in several ways. Firstly, we are considering implementing more advanced network inference methods such as EleMi [82]. Secondly, we want to implement different subcommunity detection algorithms as alternatives to *k*-means (e.g. the Leiden algorithm [83]). Finally, we want to provide an option for the user to input functional assignments of the OTUs/ASVs (such as the FUNGuild [84] output for ITS sequences). In its current state, SpeSpeNet focuses on the interaction between the microbiome and the environment. However, the three-way interaction between the environment, the microbiome, and microbial functions would be even more informative, and could reveal additional relevant biology.

In summary, SpeSpeNet speeds-up and facilitates discovery in microbial ecology by enabling researchers to interactively explore their datasets through network visualization and clustering. SpeSpeNet puts particular emphasis on the detailed exploration of potential environmental drivers of microbial community composition, either as a whole or at the level of co-abundance clusters. For these reasons, we believe that SpeSpeNet will be a valuable addition to the field of microbial ecology.

## Supplementary Material

Supplementary_materials_ycaf036

Supplementary_text_S1_ycaf036

## Data Availability

SpeSpeNet is available as a webtool from https://tbb.bio.uu.nl/SpeSpeNet. The code for SpeSpeNet (which can be used to launch SpeSpeNet offline), the code to make all figures in the manuscript and code for making networks visualizations in R using tidygraph objects downloaded from SpeSpeNet is on github: https://github.com/SnoekLab/SpeSpeNet. The data of the three case studies are available in the SpeSpeNet webtool (underneath “Select input type” choose “Example data”) and on the github as .txt files, formatted to be SpeSpeNet compatible. The SpeSpeNet manual can be found on the github and as Supplemental text S1.
