# Supplementary material for: SpeSpeNet: an interactive and user-friendly tool to create and explore microbial correlation networks
Source: ISME Commun. 2025 Feb 24;5(1):ycaf036. doi: 10.1093/ismeco/ycaf036 (PMC12341876; doi:10.1093/ismeco/ycaf036)
Supplement: Supplementary_materials_ycaf036 [file supplementary_materials_ycaf036.pdf]

Supplementary Materials

Supplementary figures

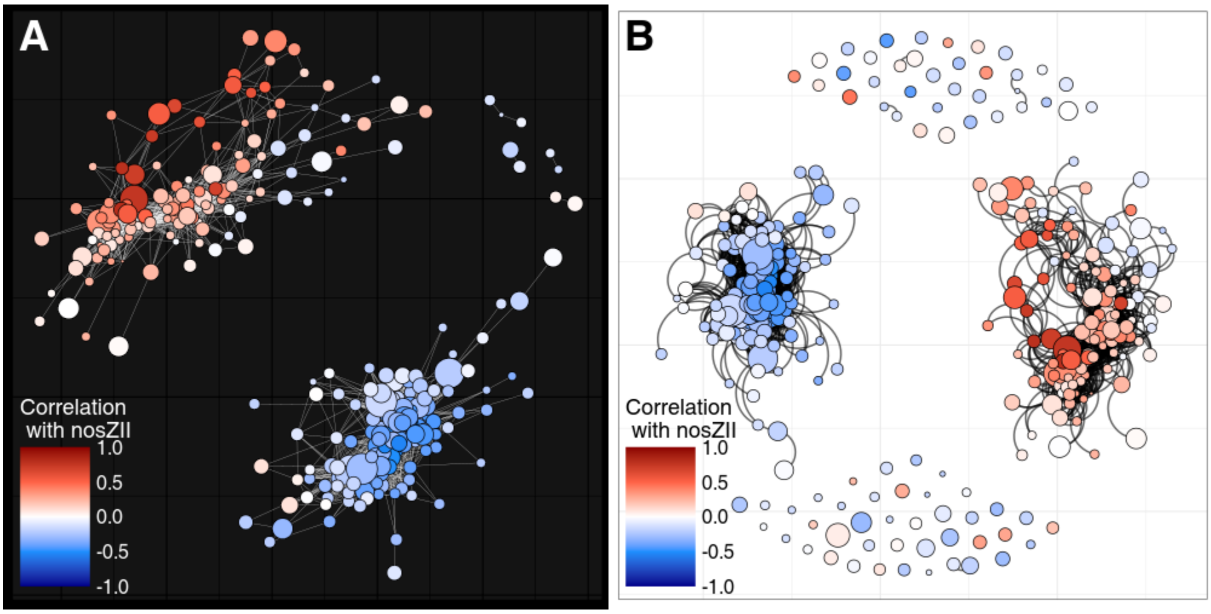

Figure S1: To illustrate customization options, we displayed the same correlation network with different aesthetic options (data from Brenzinger *et al*, same subnetwork as in Figure 4C-I). Color shows for each node (genus rank) the correlation between its CLR-transformed abundance and the qPCR abundance of the *nosZII* gene. **A)** Network with default parameters (theme = “Dark”, edge strength = 0, edge alpha = 1, edge width = 0.1, random seed = 37, not showing isolated nodes). **B)** Same network with custom aesthetic options (theme = “Classic”, edge strength = 0.8, edge alpha = 0.6, edge width = 0.7, random seed = 16, showing isolated nodes).

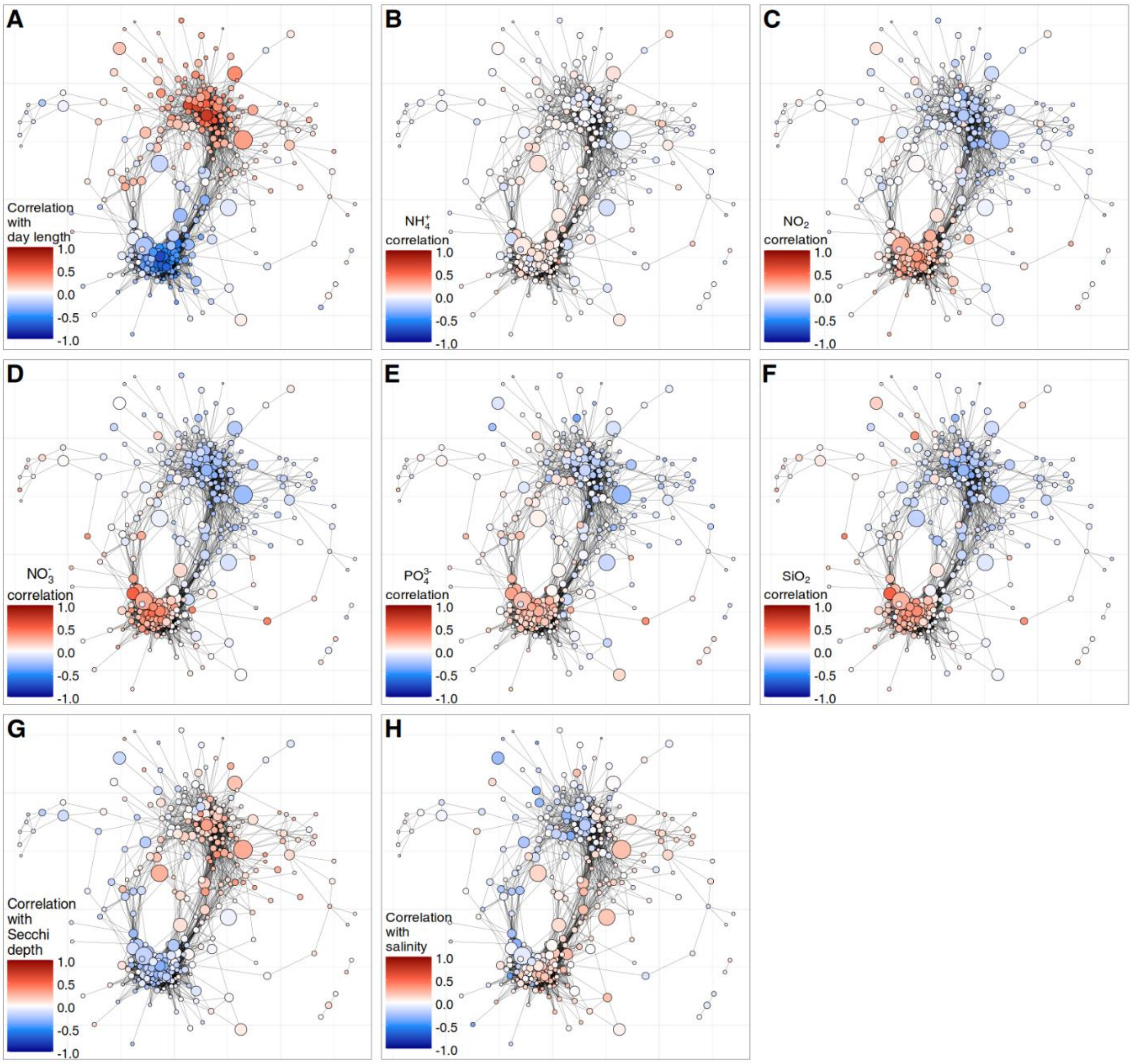

Figure S2: Inter-kingdom associations in a 10-year longitudinal study in the north-western Mediterranean Sea(44)**.** Abundance table made by merging 16S and 18S table. Same network as in **Figure 3C-F**. Nodes are genera and edges are Spearman correlations > 0.37. Networks are colored by correlation between relative abundance of genera and: **A)** Hours of daylight **B)** NH_4^+ levels **C)** NO_2 levels **D)** NO_3^- levels **E)** PO_4^{3-} levels **F)** SiO_2 levels **G)** Secchi depth (measure of water turbidity). **H)** salinity.

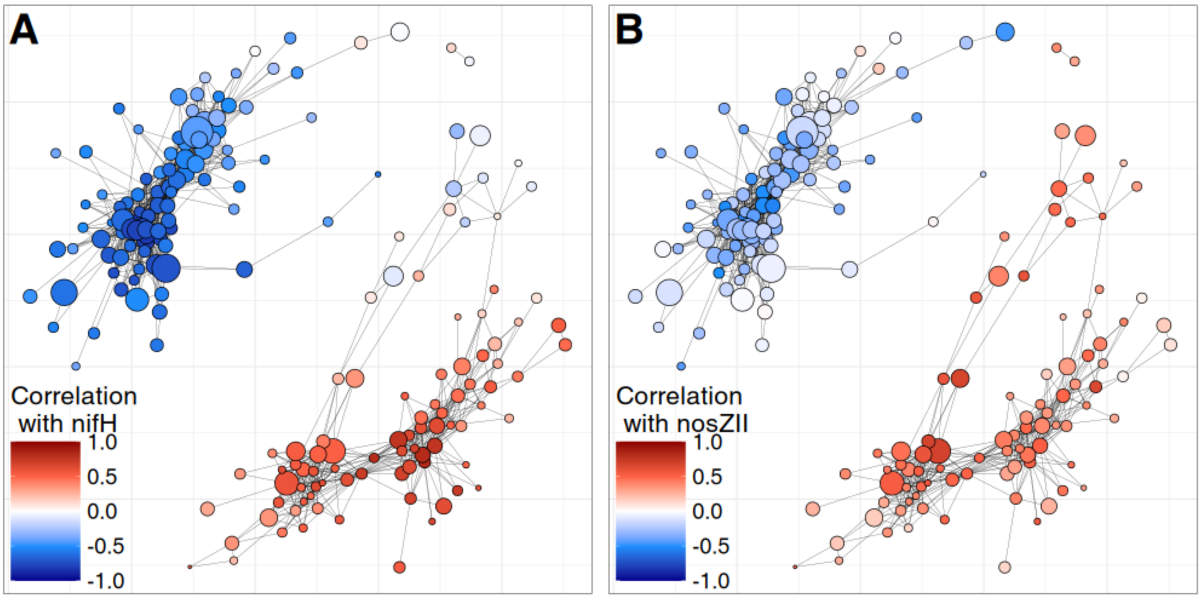

Figure S3: Correlations between genera and functional gene abundance for clay samples from Brenzinger *et al*. A) Network colored by Pearson correlation between genera and the qPCR abundance of the *nifH* gene. **B)** Network colored by Pearson correlation between genera and the qPCR abundance of the *nosZII* gene.

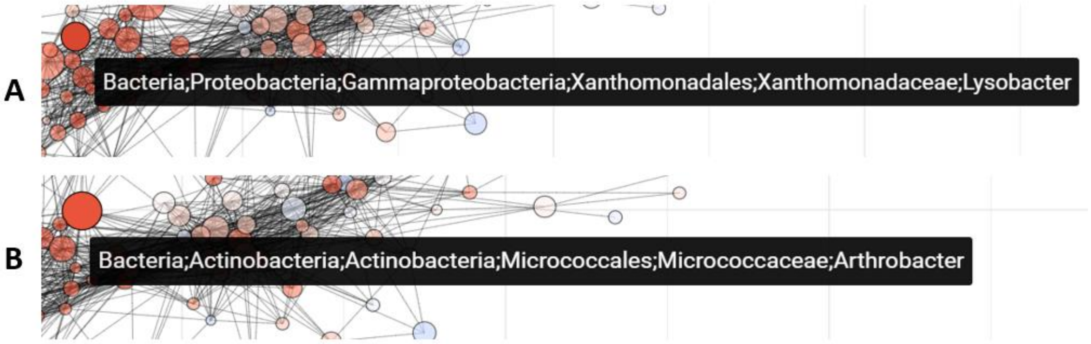

Figure S4: Mousing over the subnetwork with clay samples from Brenzinger *et al* reveals known plant-beneficial bacteria correlated with plant height. Color indicates correlation between the CLR-transformed abundance of genera and the height of plants grown on the same plot. **A)** Mousing over a genus strongly correlated with plant height shows the taxonomy (genus *Lysobacter*). **B)** Mousing over a genus strongly correlated with plant height shows the taxonomy (genus *Achromobacter*).

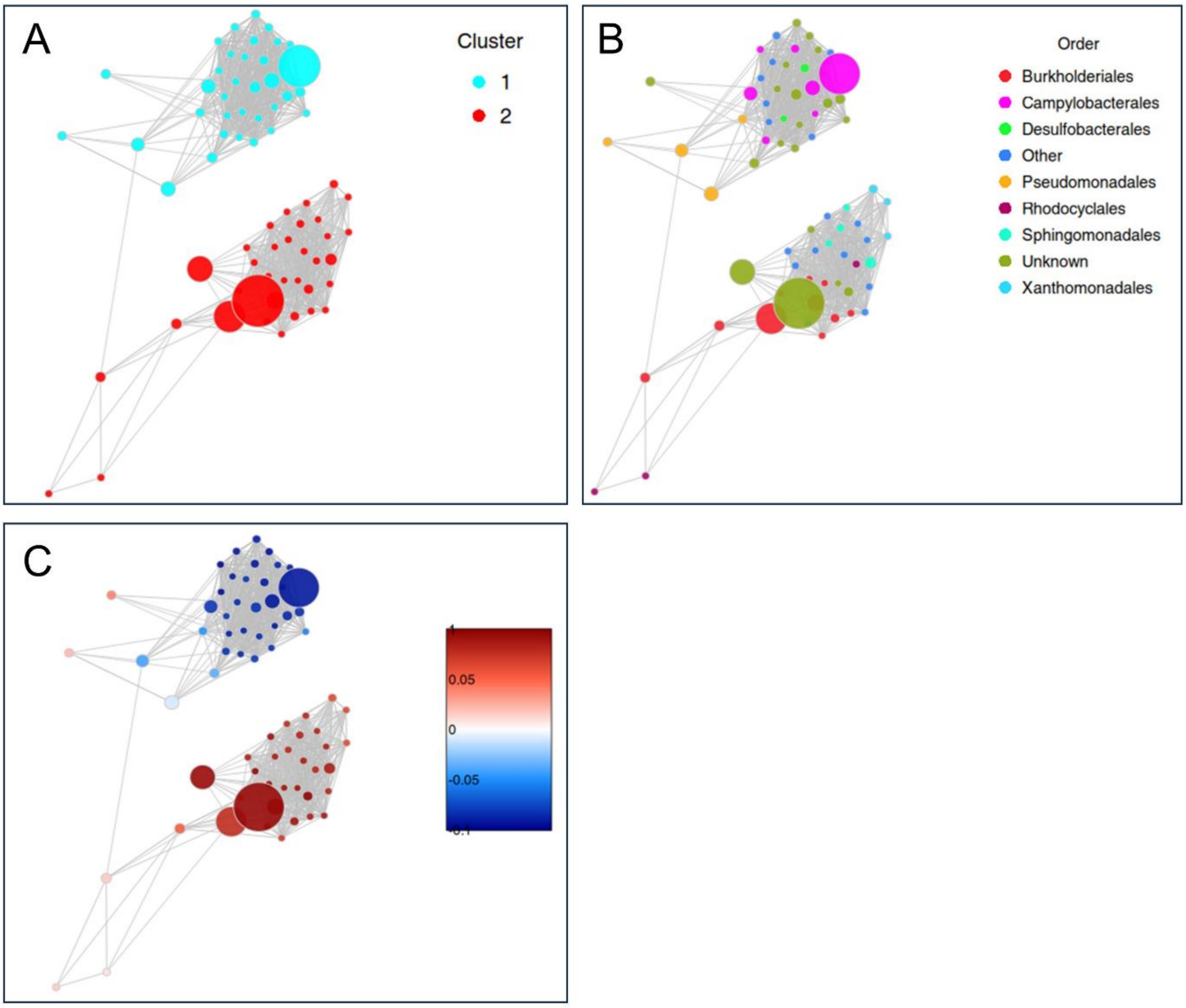

Figure S5: NetCoMi visualization of case study 1. The aim was to compare the functionalities of SpeSpeNet and NetCoMi. The network was made with similar parameters as **Figure 2**: Taxa were filtered with an occurrence threshold of five samples, zeros were substituted with pseudo-counts, and correlations were inferred using the SparCC algorithm with a threshold of 0.5. **A)** Clustering of the microbiome using the cluster_fast_greedy() algorithm from the igraph package. The SpeSpeNet version is shown in **Figure 2D**. Both NetCoMi and SpeSpeNet were able to capture the two clusters of taxa. **B)** Order rank taxonomy plotted onto nodes as in **Figure 2A**. Making this plot required us to calculate the relative abundances of all orders, select the most abundant orders, and construct a vector where the less abundant orders were labelled as "Other". **C)** Correlation with O_2 levels plotted onto nodes as in **Figure 2B**. Making this plot required us to manually calculate all center log transformed abundances, add pseudo counts, correlate abundances with the environmental variable, match every correlation value to a manually defined color scale and add a legend of the color scale to the plot.

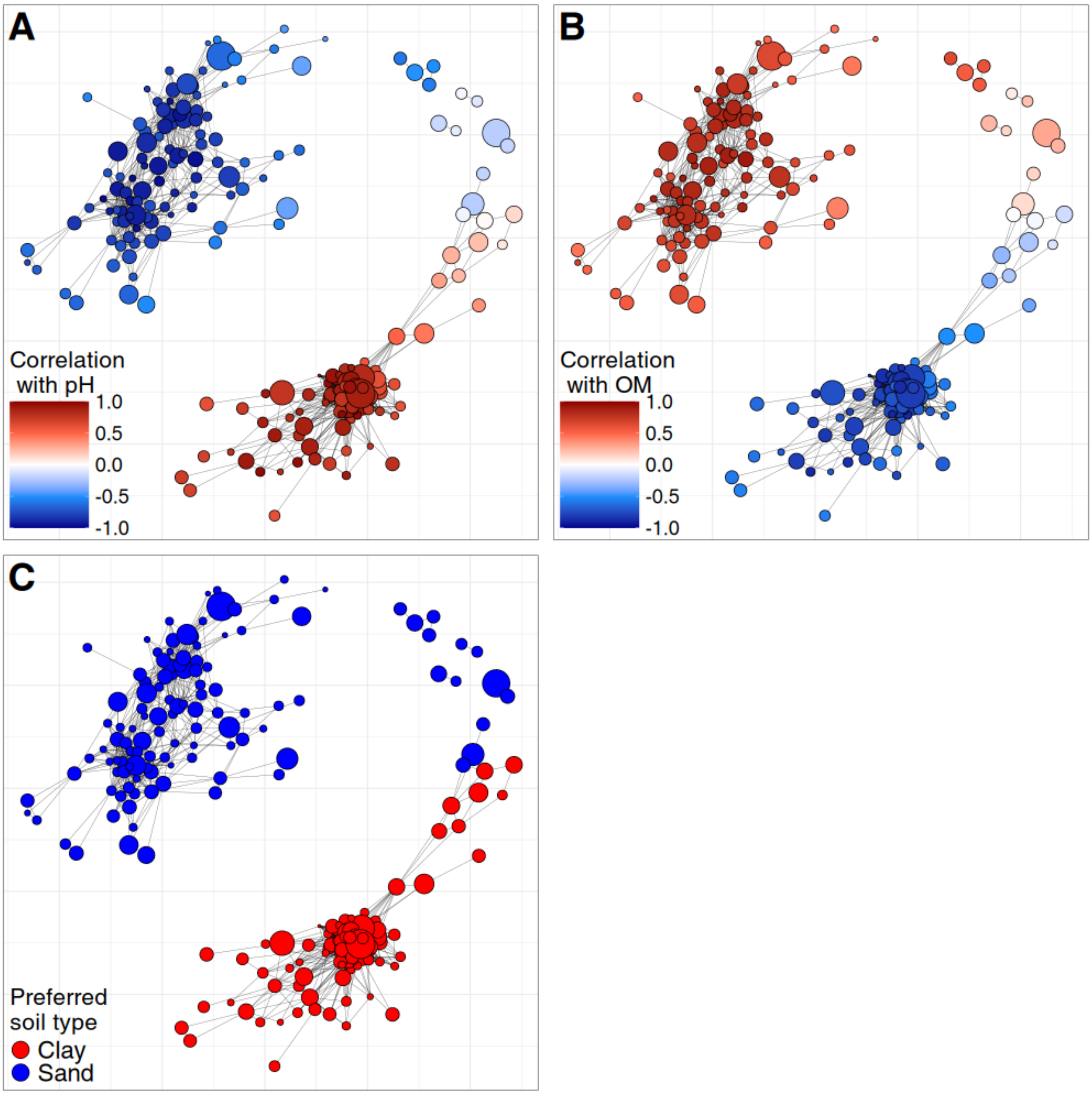

Figure S6: Correlated environmental data can lead to visually convincing patterns but a causal effect on the microbiome cannot be concluded. Same network as in **Figure 4A-B. A)** Network colored by correlation of genus CLR-transformed abundance with pH. **B)** Network colored by correlation of genus CLR-transformed abundance with organic matter (OM). **C)** Network colored by preferred soil type of genera. A researcher seeing only panel A might be convinced the pH is structuring the microbiome. However, the pH in this dataset is strongly dependent on the soil type (mean and sd of pH in sand samples is 5.646, 0.183, mean and sd of pH in clay samples is 7.559, 0.084). Furthermore, the pH has a strong inverse correlation with the organic matter content of the soil (Pearson correlation = -0.91). Hence, whether and to what extent the pH, organic matter content, particle size of the soil or a combination thereof caused the observed microbiome structure cannot be determined from these networks.
